# Retrospective analysis of genetic abnormalities and survival in 131 patients with multiple myeloma

**DOI:** 10.3892/ol.2014.2750

**Published:** 2014-12-01

**Authors:** NIAN LIU, HEBING ZHOU, GUANGZHONG YANG, CHUANYING GENG, YUAN JIAN, HUAN GUO, WENMING CHEN

**Affiliations:** Department of Hematology, Beijing Chaoyang Hospital, Capital Medical University, Beijing 100020, P.R. China

**Keywords:** multiple myeloma, fluorescence *in situ* hybridization, genetic abnormalities, prognostic factors

## Abstract

Genetic abnormalities in patients with multiple myeloma (MM) are important risk factors in terms of prognosis. In the present study, the prognostic value of several common MM genetic abnormalities was investigated. Interphase fluorescence *in situ* hybridization (iFISH) was used to detect genetic abnormalities, including 1q21 gain, t(4;14), t(11;14), t(14;16) and 17p13 deletion in 131 patients. A total of 46.6% patients were detected with one or more abnormalities using iFISH analysis. The 1q21 gain, t(4;14), t(11;14), t(14;16) and 17p13 deletion abnormalities were detected in 42.5, 6.9, 17.5, 0.8 and 10.7% of patients, respectively. Patients with t(4;14) commonly exhibited lower levels of albumin and hemoglobin. The progression-free survival (PFS) and overall survival times of iFISH-positive patients (particularly patients with two or more iFISH abnormalities) were significantly shorter than those of the patients without detectable abnormalities. The 1q21 gain and 17p13 deletion were also adverse prognostic factors for MM. Bortezomib-based therapies improved the PFS times in the patients with unfavorable iFISH abnormalities. These findings demonstrate that patients with two or more iFISH abnormalities, a gain of the 1q21 region or a 17p13 deletion were more likely to have a poor prognosis; however, bortezomib treatment improved the outcome for MM patients with unfavorable iFISH abnormalities.

## Introduction

Multiple myeloma (MM) is characterized by the clonal proliferation of plasma cells and the subsequent accumulation of these cells within the bone marrow. The prognosis of patients with MM is variable, with survival times ranging between a few months and >10 years (1). Despite the recent application of novel drugs in clinical practice, MM remains incurable. Studies with large samples have shown that molecular cytogenetic changes have an important role in the prognosis of MM (2,3). MM patients with extra copies of 1q, del(13)(q14), t(4;14), t(14;16) or del(17)(p13) exhibit an unfavorable prognosis, while the t(11;14) translocation is associated with a better outcome. The incidence rates of the aforementioned genetic abnormalities are as follows: 28.9–43.0% for 1q21 gain (4,5); 48.0–49.6% for del(13)(q14) (6,7); 11.0–17.0% for t(4;14) (6,8–13); 3.0–4.6% for t(14;16) (9,12,13); 9.5–33.8% for del(17)(p13) (6,9,11,13–15); and 12.8–21.0% for t(11;14) (6,9–12). Approximately a decade ago, combination chemotherapy regimens, such as melphalan-prednisone or vincristine-doxorubicin-dexamethasone combined with stem cell transplantation were the standard treatment modalities for MM. However, the introduction of newer therapies, including the proteasome inhibitor bortezomib and immunomodulatory drugs (thalidomide and lenalidomide) has significantly improved patient survival. Previous studies have also shown that bortezomib induction improves the outcome of newly diagnosed patients with t(4;14) (16–18). The main objective of the present study was to evaluate the frequency and prognostic impact of several common fluorescence *in situ* hybridization (iFISH) abnormalities in patients with MM.

## Patients and methods

### Patients

A total of 107 newly diagnosed patients and 24 relapsed MM patients from Beijing Chaoyang Hospital (Beijing, China) were included in the present study. The diagnostic criteria were primarily derived from those provided by the World Health Organization (19). The International Staging System (ISS) (20) and the Durie-Salmon (DS) staging (21) were employed to assess these patients. Among the 128 patients with available treatment information, 88 received bortezomib-based combination chemotherapy. This treatment was composed of bortezomib (1.3 mg/m^2^, days 1, 4, 8 and 11) plus dexamethasone (20 mg/day, days 1, 2, 4, 5, 8, 9, 11 and 12) or dexamethasone in combination with doxorubicin (9 mg/m^2^, days 1–4), Cytoxan (300 mg/m^2^, days 1–4) or thalidomide (100 mg/day, days 1–21). The patients who received this regimen were administered a median of four treatment courses (1–7 three-week courses). The other 40 patients received non-bortezomib-based therapy, involving either doxorubicin (9 mg/m^2^, days 1–4), vincristine (0.4 mg/day, days 1–4) and dexamethasone (20 mg/day, days 1–4, 9–12 and 17–20), thalidomide (100 mg/day, day 1–28), doxorubicin (9 mg/m^2^, days 1–4) and dexamethasone (20 mg/day, days 1–4, 9–12 and 17–20), or melphalan (4 mg/m^2^, days 1–7), prednisone (60 mg/day, days 1–7) and thalidomide (100 mg/day, days 1–28). The patients on this regime received a median of four treatment courses (1–8 four week courses). Following the induction of therapy, 23 patients received autologous stem cell transplantation and maintenance therapy, and the other 108 patients received only maintenance therapy. The efficacy assessment was conducted according to the International Myeloma Working Group criteria (22). The median follow-up time was 21.3 months (range, 0.2–109.3 months). The clinical and laboratory features of all 131 patients are summarized in Table I. This study was approved by the ethics committee of Beijing Chaoyang Hospital and written informed consent was obtained from all patients.

### Interphase FISH (iFISH) analysis

A 5–10-ml sample of bone marrow was obtained with informed consent from the patients with MM at the time of diagnosis or relapse, and was then mixed with heparin. Mononuclear cells were enriched by the Ficoll-gradient centrifugation method (Ficoll-Paque PLUS; GE Healthcare Bio-Sciences AB, Uppsala, Sweden), and then were assessed using commercially available probes for the regions containing 4p16 (*FGFR3*)/14q32 (*IGH*), 11q13 (*CCND1*)/14q32 (*IGH*), 14q32 (*IGH*)/16q32 (*MAF*), 1q21 and 17p13.1 (*TP53*) using Vysis TP53/CEP 17 FISH, Vysis IGH/MAF DF FISH and Vysis IGH/CCND1 DF FISH Probe Kits, a Vysis LSI IGH/FGFR3 Dual Color, Dual Fusion Translocation Probe (Vysis/Abbott Molecular, Des Plaines, IL, USA) and a LSI 1q21 FISH Probe Kit (China Meditech, Beijing, China). Slides containing the cells were pretreated, denatured and hybridized using standard laboratory procedures following the manufacturer’s instructions (Vysis/Abbott Molecular). A minimum of 500 mononuclear cells were scored for each iFISH signal to estimate the prevalence of each molecular cytogenetic abnormality. The positive cutoff level for each iFISH probe set was established as >10%.

### Statistical analysis

Associations between genetic abnormalities and biological parameters were evaluated by independent samples t-test. Comparison of frequencies among groups was performed using the χ^2^ test. The overall survival (OS) and progression-free survival (PFS) time distributions were estimated using the Kaplan-Meier method, and differences between survival curves were analyzed using the log-rank test. PFS times were only evaluated in patients who achieved at least a partial response (PR). All statistical analyses were performed using SPSS version 18.0 (SPSS, Inc., Chicago, IL, USA). P<0.05 was considered to indicate a statistically significant difference.

## Results

### Genetic abnormalities detected by iFISH

A total of 61 patients (46.6%) exhibited abnormal iFISH results. Gain of 1q21 was the most common genetic abnormality, with an incidence of 42.5% (34/80), whereas t(14;16) was the least common abnormality exhibited, with an incidence of 0.8% (1/131). The presence of the 17p deletion was detected in 10.7% (14/131) of cases. A total of 17.5% (14/80) of patients had t(11;14), whereas 6.9% (9/131) had t(4;14). Two cases (2.5%) presented with three abnormalities, 18 cases (22.5%) with two and 27 cases (33.8%) with one. In total, of the 80 patients tested, 33 patients (41.3%) did not exhibit any of the five abnormalities. Due to the small sample size, t(14;16) was not included in any further analysis.

### Correlation between iFISH abnormalities and biological parameters

No significant differences in gender (P=0.648), age (≥65 years vs. <65 years; P=0.586) and status of disease (newly diagnosed or relapsed; P=0.709) were identified between patients with positive iFISH results and those without an abnormality. However, in comparison to patients with normal iFISH results, the patients with abnormal iFISH results exhibited significantly higher calcium levels (median, 2.26 vs. 2.14 mmol/l; P=0.002), lower hemoglobin levels (median, 8.8 vs. 9.7 g/dl; P=0.024) and a higher DS stage (P=0.018). β2-microglobulin and C-reactive protein levels were also higher in the patients with abnormal iFISH results compared with the patients with normal iFISH results (median, 5.09 vs. 3.06 mg/l and 0.50 vs. 0.42 mg/l, respectively), but these results were not statistically significant. In addition, in comparison to patients who lacked t(4;14), the patients with t(4;14) had significantly lower albumin levels (median, 25.0 vs. 32.6 g/l; P=0.008) and lower hemoglobin levels (median 7.6 vs. 9.5 g/dl; P<0.001).

### Correlation between iFISH abnormalities and response rates

A total of 122 patients had best treatment response assessment results available. The overall response rate (ORR; PR or better) of treatment was 84.4% (103/122) and a very good partial response (VGPR) or better was achieved in 42.6% (52/122) patients. The 65 patients who had no detectable abnormalities exhibited an ORR of 86.2% (56/65), a rate comparable with that of patients with iFISH abnormalities (82.5%; 47/57). Similarly, no significant differences in ORR were observed between the patients with versus those without del(17p) (75.0 vs. 85.5%; P=0.597), t(11;14) (69.2 vs. 82.0%; P=0.511), t(4;14) (87.5 vs. 84.2%; P=1.000) and 1q21 gain (82.4 vs. 77.5%; P=0.605), respectively. These results indicate that the treatment response of the patients with MM was not affected by the presence/absence of the iFISH abnormalities under investigation.

However, in the iFISH-negative group, the patients who received bortezomib-based combination chemotherapy exhibited a significantly higher ORR (95.3 vs. 68.2%; P=0.009) and a higher ≥VGPR (60.1 vs. 13.6%; P=0.001) compared with the patients who received the non-bortezomib regimens. Furthermore, for the patients with t(4;14), the bortezomib-based combination improved the rate of ≥VGPR (100.0 vs. 0.0%; P=0.001; Table II).

### Correlation between iFISH abnormalities and patient outcome

**Figure 1:**
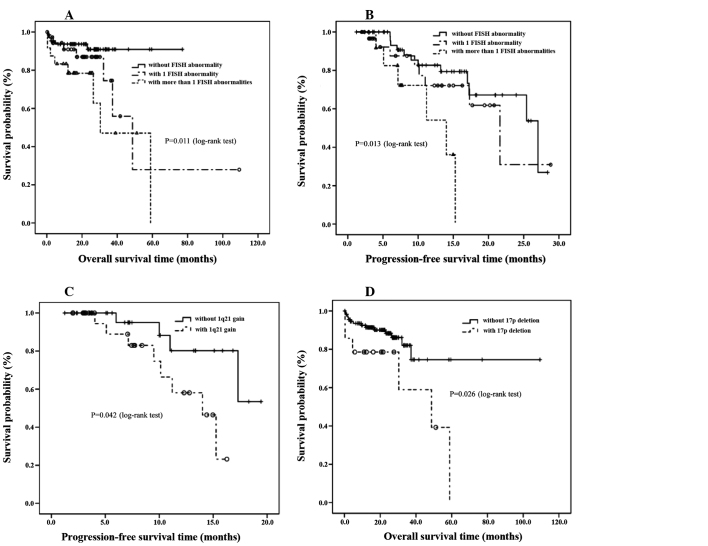
(A) Overall survival (OS) times of multiple myeloma (MM) patients according to fluorescence *in situ hybridization* (FISH) abnormalities. (B) Progression-free survival (PFS) times of MM patients according to FISH abnormalities. (C) PFS times of MM patients according to 1q21 gain. (D) OS times of MM patients according to 17p deletion.

The prognostic impact of each iFISH abnormality on the PFS and OS times was analyzed. Patients with two or more iFISH abnormalities had significantly shorter OS and PFS times than patients with one or no abnormality (median OS time, 30.4 vs. 48.7 months vs. not reached (OS time>follow-up time), respectively; P=0.013; Fig. 1A; and median PFS time, 14.0 vs. 21.6 vs. 27.0 months, respectively; P=0.011; Fig. 1B). Significantly shorter PFS times were observed in the patients with 1q21 gain versus those without 1q21 gain (median PFS time, 14.0 months vs. not reached, respectively; P=0.042; Fig. 1C). The patients with the 17p13 deletion had a significantly poorer outcome compared with the patients without the 17p13 deletion, with a median OS time of 48.7 months versus not reached, respectively (P=0.026; Fig. 1D). The presence/absence of t(11;14) and t(4;14) were not associated with a statistically significant effect on the PFS or OS times. All data are summarized in Table III.

Of the patients administered non-bortezomib-based chemotherapy, those with two or more iFISH abnormalities had shorter PFS and OS times than those with one or no abnormality (median PFS time, 7.1 vs. 21.6 vs. 27.0 months, respectively; P=0.034; and median OS time, 30.4 vs. 48.7 months vs. not reached, respectively; P=0.245). For the patients who received bortezomib-based regimens, the differences between PFS times for those with two or more iFISH abnormalities and those with one abnormality or no abnormality were not statistically significant (median PFS times, 15.3 months vs. not reached vs. not reached, respectively, P=0.237; median OS times for the three groups were 37.3, 58.9 months and not reached, respectively; P=0.225). Thus, bortezomib appears to partially overcome the adverse effect caused by iFISH abnormalities.

## Discussion

The present retrospective study included a series of 131 patients with MM who were analyzed for genomic aberrations and were treated with bortezomib/non-bortezomib-based therapies. The incidence of translocation t(4;14) in the present study (6.9%) was marginally lower than that reported by Fonseca *et al* (12.7%) (9) and that observed by Avet-Loiseau *et al* (14%) (6). The incidence of t(14;16) in the present study was rare, detected in only one case out of 131 myeloma patients, which may be due to the small sample size. The observed frequencies of 1q21 gain, t(11;14) and 17p13 deletion in the present study were consistent with results published in previous studies (6,9,23). Of the 61 patients with abnormal iFISH results in the present study, 24 (39.3%) had two or more iFISH abnormalities. These results reflect the fact that myeloma cells are characterized by complex genetic abnormalities.

The most extensively employed prognostic system in myeloma is the ISS, a system which stratifies patients into three groups, as determined by serum albumin and β2-microglobulin levels. These are factors that are reflective of patient and tumor characteristics, with β2-microglobulin an indicator of tumor bulk and renal function, and albumin associated with the patient’s general state. In the present study, the ISS stage was not affected by the presence or absence of any known iFISH abnormality, although patients with t(4;14) had significantly lower albumin levels than patients who lacked t(4;14).

ORR analysis did not reveal statistical differences between the patients with and without iFISH abnormalities. However, in the patients with normal iFISH results, bortezomib-based therapies significantly improved the ORR (95.3 vs. 68.2%) and ≥VGPR (60.1 vs. 13.6%) compared with the patients receiving non-bortezomib-based therapies. However, for the patients with iFISH abnormalities, bortezomib-based therapies were not shown to be more effective, with the exception that these therapies improved the ≥VGPR (100.0 vs. 0.0%) for the patients with t(4;14).

In the present study, the presence of two or more iFISH abnormalities remained the most important unfavorable prognostic factor for patients with MM. The median PFS time of the patients with two or more iFISH abnormalities was found to be only 14.0 months and the OS time reached only 30.4 months; a finding consistent with that of a previous study, which demonstrated that patients with complex karyotypes had the worst outcome (23). The gain of 1q21 has been reported to be an adverse prognostic factor in newly diagnosed or relapsed MM in several studies (4,5,24). In the present study, 1q21 gain, examined by interphase iFISH in 80 patients with myeloma, exerted a marked effect on prognosis. Gain of the 1q21 locus reduced the PFS time to 14.0 months, the same time period as that found in the patients with two or more iFISH abnormalities. However, 1q21 gain was not confirmed as a prognostic factor for OS time (P=0.885). Deletion and mutation of the p53 tumor suppressor gene, located in 17p13, are commonly observed in patients with MM. The 17p13 deletion is detected in ~10% patients with MM at the time of diagnosis and this abnormality frequently occurs together with other aberrations (25). Mutation and deletion of the p53 tumor suppressor gene have been markedly associated with a lower likelihood of a response to therapy, and have been revealed as independent prognostic markers for shorter survival times in MM patients (16,25–27). In the present study, which sampled 131 MM cases, patients with the 17p13 deletion exhibited a significantly shorter OS time than patients without this abnormality (48.7 months vs. not reached; P=0.026). Furthermore, out of the 14 patients with the 17p13 deletion, six (42.9%) also had a 1q21 gain. However, the prognosis in this group was not significantly different compared with the prognosis of patients who only had the 17p13 deletion. Other genomic changes, such as t(4;14) and t(11;14), had less effect on PFS and OS times. Previous studies have reported that t(4;14) is an unfavorable prognostic factor (20,28). The results of the present study suggest that patients with t(4;14) had a shorter PFS and OS times than patients who lacked t(4;14) (PFS, 17.3 vs. 25.4 months; OS, 58.9 months vs. not reached); however, this difference was not statistically significant. The lack of a statistically significant difference may be due to the heterogeneity of t(4;14), or the limited sample size and low number of follow-ups in the present study. Similarly, t(11;14) did not affect the survival outcome, as suggested by several other studies (6,29,30).

Previous clinical trials have demonstrated that bortezomib-based regimens are effective in the treatment of MM and are able to overcome the unfavorable effect of several abnormalities [del (13q), t(4;14), t(14;16), del (p53) and 1q21 gain] (31–35). In the present study, the patients with two or more iFISH abnormalities had significantly shorter PFS times than the patients with less or no iFISH abnormality following administration of non-bortezomib-based regimens. However, when the patients received bortezomib-based regimens, the PFS times of the patients with two or more iFISH abnormalities, one abnormality and no abnormality were not significantly different. Thus, bortezomib may improve the adverse outcomes caused by iFISH abnormalities.

In conclusion, the present study demonstrated that patients with two or more iFISH abnormalities, gain of the 1q21 region or deletion of 17p13 were more likely to exhibit a poor prognosis for MM, and that bortezomib improves the outcome for MM patients with unfavorable iFISH abnormalities. Due to the limited sample size and follow-ups, larger prospective trials are required for a more reliable result.

## Figures and Tables

**Table I:** Characteristics of patients.

Characteristics	Total no. of patients	Value
Median age, years (range)		59 (38–82)
Gender, n (%)	131	
Male		74 (56.5)
Female		57 (43.5)
Durie-Salmon stage, n (%)	114	
I		1 (0.9)
II		15 (13.1)
III		98 (86.0)
Durie-Salmon substage, n (%)	109	
A		75 (68.8)
B		34 (31.2)
ISS stage, n (%)	105	
I		11 (10.5)
II		39 (37.1)
III		55 (52.4)
Ig isotype, n (%)	129	
IgG		59 (45.7)
IgA		33 (25.6)
IgD		9 (7.0)
Light chain		23 (17.8)
No paraprotein		5 (3.9)
Median Hb level, g/dl (range)	124	9.3 (4.4–16.6)
Median calcium level, mmol/l (range)	124	2.18 (1.25–3.83)
Median CRP level, mg/l (range)	94	0.4 (0.1–13.7)
Median β2-microgolbulin level, mg/l (range)	124	3.50 (0.95–34.15)
Median albumin level, g/l (range)	124	31.9 (15.2–46.5)
Treatment response, n (%)	122	
CR		36 (29.5)
VGPR		16 (13.1)
PR		51 (41.8)
<PR		19 (15.6)
Treatment, n (%)	128	
Bortezomib-based combination chemotherapy		88 (68.8)
Non-bortezomib-based therapy		40 (31.2)
ASCT, n (%)	131	
Yes		23 (17.6)
No		108 (82.4)

ISS, International Staging System; Hb, hemoglobin; CRP, C-reactive protein; CR, complete response; VGPR, very good partial response; PR, partial response; ASCT, autologous stem cell transplantation.

**Table II:** Response rate of multiple myeloma patients receiving bortezomib-based therapy or conventional therapy.

	ORR, n (%)		≥VGPR, n (%)	
		
	Bortezomib-based therapy	Non-bortezomib-based therapy	Bortezomib-based therapy	Non-bortezomib-based therapy
Normal FISH	41 (95.3)	15 (68.2)	26 (60.1)	3 (13.6)
del(17)(p13)	6 (75.0)	3 (75.0)	4 (50.0)	1 (25.0)
1q21 gain	20 (87.0)	8 (72.7)	11 (47.8)	2 (18.2)
t(4;14)(p16;q32)	4 (100.0)	3 (75.0)	4 (100.0)	0 (0.0)
t(11;14)(q13;q32)	7 (70.0)	2 (66.7)	1 (10.0)	1 (33.3)

ORR, overall response rate; VGPR, very good partial response; FISH, fluorescence *in situ* hybridization.

**Table III:** PFS and OS of multiple myeloma patients according to cytogenetic abnormalities.

		PFS time			OS time		
			
	n	Median, months	Three-year estimate, % (mean ± SD)	P-value	Median, months	Three-year estimate, % (mean ± SD)	P-value
Overall				0.011			0.013
2 or more abnormalities	24	14.0	0.0±0.0		30.4	47.1±17.9	
1 abnormality	37	21.6	30.9±22.8		48.7	74.5±12.7	
Normal FISH	66	27.0	62.7±9.7		NR	90.9±4.0	
t(11;14)				0.839			0.289
Positive	14	15.3	0.0±0.0		30.4	38.1±27.6	
Negative	66	17.3	39.9±17.8		NR	75.1±9.6	
t(4;14)				0.975			0.588
Positive	9	17.3	46.7±22.6		58.9	59.3±25.2	
Negative	118	25.4	76.5±5.4		NR	90.0±2.9	
17p deletion				0.760			0.026
Positive	14	21.6	0.0±0.0		48.7	58.9±18.9	
Negative	113	25.4	31.7±14.9		NR	91.4±2.7	
1q21 gain				0.042			0.885
Positive	34	14.0	23.2±18.1		30.4	44.9±21.3	
Negative	46	NR	53.5±22.9		NR	79.8±8.0	

PFS, progression-free survival; OS, overall survival; FISH, fluorescence *in situ* hybridization; NR, not reached; SD, standard deviation.
